# Dihydroxyacetone metabolism in *Haloferax volcanii*

**DOI:** 10.3389/fmicb.2013.00376

**Published:** 2013-12-16

**Authors:** Matthew Ouellette, Andrea M. Makkay, R. Thane Papke

**Affiliations:** Department of Molecular and Cell Biology, University of ConnecticutStorrs, CT, USA

**Keywords:** dihydroxyacetone metabolism, dihydroxyacetone kinase, glycerol kinase, archaea, Halobacteria, Haloarchaea

## Abstract

Dihydroxyacetone (DHA) is a ketose sugar that can be produced by oxidizing glycerol. DHA in the environment is taken up and phosphorylated to DHA-phosphate by glycerol kinase or DHA kinase. In hypersaline environments, it is hypothesized that DHA is produced as an overflow product from glycerol utilization by organisms such as *Salinibacter ruber*. Previous research has demonstrated that the halobacterial species *Haloquadratum walsbyi* can use DHA as a carbon source, and putative DHA kinase genes were hypothesized to be involved in this process. However, DHA metabolism has not been demonstrated in other halobacterial species, and the role of the DHA kinase genes was not confirmed. In this study, we examined the metabolism of DHA in *Haloferax volcanii* because putative DHA kinase genes were annotated in its genome, and it has an established genetic system to assay growth of mutant knockouts. Experiments in which *Hfx. volcanii* was grown on DHA as the sole carbon source demonstrated growth, and that it is concentration dependent. Three annotated DHA kinase genes (HVO_1544, HVO_1545, and HVO_1546), which are homologous to the putative DHA kinase genes present in *Hqm. walsbyi*, as well as the glycerol kinase gene (HVO_1541), were deleted to examine the effect of these genes on the growth of *Hfx. volcanii* on DHA. Experiments demonstrated that the DHA kinase deletion mutant exhibited diminished, but not absence of growth on DHA compared to the parent strain. Deletion of the glycerol kinase gene also reduced growth on DHA, and did so more than deletion of the DHA kinase. The results indicate that *Hfx. volcanii* can metabolize DHA and that DHA kinase plays a role in this metabolism. However, the glycerol kinase appears to be the primary enzyme involved in this process. BLASTp analyses demonstrate that the DHA kinase genes are patchily distributed among the Halobacteria, whereas the glycerol kinase gene is widely distributed, suggesting a widespread capability for DHA metabolism.

## Introduction

Dihydroxyacetone (DHA) is a simple ketose sugar commonly used in sunless tanning lotions and sprays (Faurschou et al., 2004). DHA can be used as a carbon source by many different bacteria, yeast, and protists, and there are a number of different pathways in which it can be produced. In bacteria such as *Klebsiella pneumoniae*, DHA is produced anaerobically via glycerol oxidation by an NAD-dependent glycerol dehydrogenase (Forage and Lin, 1982). *Gluconobacter oxydans* and related bacteria also use glycerol oxidation to produce DHA, but they utilize a glycerol dehydrogenase that is pyrroloquinoline quinone (PQQ)-dependent and attached to the outer membrane. This pathway releases the DHA directly into the surrounding environment, which makes the *Gluconobacter* bacteria useful for industrial production of DHA (Deppenmeier et al., 2002). DHA can also be produced by methylotrophic yeast such as *Candida boidinii* by first oxidizing methanol to formaldehyde, after which a pyrophosphate-dependent transketolase transfers a two-carbon hydroxyethyl group to the formaldehyde to form DHA (Waites and Quayle, 1981).

Once DHA is obtained by a cell either via glycerol oxidation or uptake from the surrounding environment, it can then be phosphorylated and subsequently metabolized. Two types of kinases phosphorylate DHA: glycerol kinase and DHA kinase. Glycerol kinase is considered less specific, and it is capable of phosphorylating both glycerol and DHA using ATP (Hayashi and Lin, 1967; Weinhouse and Benziman, 1976; Jin et al., 1982). DHA kinase is more specific, and it is only able to phosphorylate DHA and its isomer, D-glyceraldehyde (Erni et al., 2006). There are two major families of DHA kinases. The first consists of two subunits (DhaK and DhaL) and which are ATP-dependent. The DhaK subunit binds to the DHA substrate, and the DhaL subunit binds to ATP and transfers a phosphate group from ATP to DhaK-DHA (Daniel et al., 1995; Siebold et al., 2003). In the second family, the DHA kinases are made up of three subunits (DhaK, DhaL, and DhaM) and are phosphoenolpyruvate (PEP)-dependent. This family of DHA kinases uses the PEP:sugar phosphotransferase system (PTS) to transfer a phosphate group from PEP to the DhaM subunit, a multidomain protein with one domain predicted to be a member of the mannose (EIIA^Man^) family of the PTS (Gutknecht et al., 2001; Zurbriggen et al., 2008). The DhaM then transfers the phosphate group to DhaL, which picks up the phosphate using an ADP cofactor bound to the subunit (Bachler et al., 2005). The phosphate is then transferred from DhaL to the DhaK subunit, which phosphorylates the bound DHA substrate to DHA phosphate. The ATP-dependent family of DHA kinases is present in eukaryotes and some bacteria, whereas the PEP-dependent family of DHA kinases is present only in bacteria and archaea (Erni et al., 2006).

DHA has been hypothesized as a potential carbon source in hypersaline environments for heterotrophic halobacterial species (Elevi Bardavid et al., 2008). This hypothesis is supported by previous studies on glycerol oxidation in *Salinibacter ruber*, a halophilic bacterium common in hypersaline environments. In a study by Sher et al. (2004), which examined the oxidation of radio-labeled glycerol by *S. ruber*, an unknown soluble product consisting of 20% of the radioactivity from the added glycerol was observed to be excreted by the cells. This soluble product was later analyzed in a study by Elevi Bardavid and Oren (2008) using a colorimetric assay, and was identified as DHA; indicating that *S. ruber* could produce DHA in hypersaline environments as an overflow product via glycerol oxidation.

The ability of *Haloquadratum walsbyi*, a common halobacterial species, to metabolize DHA further supports the hypothesis that DHA is a carbon source in hypersaline environments. *Hqm. walsbyi* was first hypothesized to metabolize DHA after examination of the sequenced genome in a study Bolhuis et al. (2006) identified an uptake system for DHA involving three genes (HQ2672A, HQ2673A, and HQ2674A) encoding the subunits of a putative PEP-dependent DHA kinase. The DHA kinase encoded by these genes was hypothesized to use a phosphate group from the PTS system to phosphorylate DHA to DHA phosphate, which could then be incorporated into the metabolism of the cell. Elevi Bardavid and Oren (2008) tested DHA metabolism in *Hqm. walsbyi* by adding DHA to a cell culture of *Hqm. walsbyi* and measuring the change in DHA concentration over time. A decrease in DHA concentration was observed, indicating that the DHA was being taken up and metabolized by the *Hqm. walsbyi* cultures.

Overall, the current evidence supports a model where halobacterial species *Hqm. walsbyi* metabolizes DHA in hypersaline environments produced by *S. ruber*; however, there is still little known about DHA metabolism in Halobacteria. While DHA metabolism has been observed to occur in *Hqm. walsbyi*, no other halobacterial species has been shown to be able to metabolize DHA. Additionally, the putative DHA kinase genes in *Hqm. walsbyi* were never confirmed to be involved in DHA phosphorylation and metabolism. In this study, we sought to elucidate our understanding of halobacterial metabolism of DHA by examining DHA utilization in *Haloferax volcanii*, a halobacterial species isolated from Dead Sea sediment (Mullakhanbhai and Larsen, 1975). We used *Hfx. volcanii* because it has three putative PEP-dependent DHA kinase genes that are homologous to *Hqm. walsbyi* (Anderson et al., 2011), and it has an established genetic system that can be used to delete genes and test their function (Bitan-Banin et al., 2003; Allers et al., 2004; Blaby et al., 2010). We also used DHA metabolism genes in *Hfx. volcanii* to search the other sequenced halobacterial genomes to better understand the distribution of these genes among the Halobacteria. Our data provide important new insights into the metabolism of DHA in halobacterial organisms.

## Materials and methods

### Strains and growth conditions

Strains and plasmids used in this study are listed in Table 1. All *Hfx. volcanii* strains were grown in either Hv-YPC or Hv-CA medium at 42°C while shaking at 200 rpm. Hv-YPC and Hv-CA media were produced using the formulas outlined in *The Halohandbook* (Dyall-Smith, 2009). Hv-min medium used in growth experiments was modified from the formula in *The Halohandbook* to exclude a carbon source (Hv-min -C). Media were supplemented with uracil (50 μg/mL) and 5-fluoroorotic acid (50 μg/mL) as needed. For growth on Petri plates, 2% agar (w/v) was added to the media.

**Table 1:** **List of plasmids and strains used in this study**.

**Plasmid or Strain**	**Description**	**References**
pTA131	Cloning vector used for gene deletion in *Hfx. volcanii.* Contains lacZ cloning site, ampicillin resistance gene for screening in *E. coli* and *pyrE2* gene for screening in *Hfx. volcanii.*	Allers et al., 2004
pTA409	Cloning vector used for gene complementation in *Hfx. volcanii.* Contains *lacZ* cloning site, ampicillin resistance gene for screening in *E. coli* and *pyrE2* gene for screening in *Hfx. volcanii.*	Holzle et al., 2008
pΔ*dhaKLM*	Derivative of pTA131 used to delete *dhaKLM* in *Hfx. volcanii.*	This study
pΔ*glpK*	Derivative of pTA131 used to delete glpK in *Hfx. volcanii.*	This study
p*dhaKLM*	Derivative of pTA409 used to complement *dhaKLM* in Δ*dhaKLM* strain.	This study
p*glpK*	Derivative of pTA409 used to complement *glpK* in Δ*glpK* strain.	This study
HST08	An *E. coli* strain used for screening of constructed plasmids.	Clontech, Cat. # 636763
*dam*^−^/*dcm*^−^	An *E. coli* strain used to demethylate constructed plasmids.	New England BioLabs, Cat. # C2925H
H26	Uracil auxotrophic strain of *Hfx. volcanii.*	Allers et al., 2004
Δ*dhaKLM*	Derivative strain of H26 with *dhaKLM* operon deleted.	This study
Δ*dhaKLM* + p*dhaKLM*	Derivative strain of Δ*dhaKLM* with complementation of *dhaKLM* operon.	This study
Δ*glpK*	Derivative strain of H26 with *glpK* gene deleted.	This study
Δ*glpK* + p*glpK*	Derivative strain of Δ*glpK* with complementation of *glpK* gene.	This study
Δ*dhaKLM ΔglpK*	Derivative strain of H26 with *dhaKLM* operon and *glpK* gene deleted.	This study
Δ*dhaKLM ΔglpK* + p*dhaKLM*	Derivative strain of Δ*dhaKLM ΔglpK* with complementation of *dhaKLM* operon.	This study
Δ*dhaKLM ΔglpK* + p*glpK*	Derivative strain of Δ*dhaKLM ΔglpK* with complementation of *glpK* gene.	This study

All *Escherichia coli* strains were grown in either S.O.C. media or LB-media at 37°C while shaking at 200 rpm. S.O.C. media was provided by Clontech (Cat. # 636763) and New England BioLabs (Cat. # B9020S). LB medium was produced by adding 5 g NaCl, 5 g tryptone, and 2.5 g of yeast extract to deionized water to a final volume of 500 mL and pH set to 7.0. LB was supplemented with ampicillin (100 μg/mL) as needed. When LB cell culture plates were produced, 1.5% agar (w/v) was added. LB plates were supplemented with 40 μL of X-gal (20 mg/mL) as needed.

### PCR and DNA isolation

All primers used in this study are listed in Table 2. DNA used for plasmid construction and screening was amplified via PCR. Reactions for PCR were assembled as 10 μL volumes and contained the following reagents: 5.9 μL of deionized water, 2 μL of 5x GC Phusion buffer (Thermo Scientific, Cat. # F-519), 1 μL of 100% DMSO (Thermo Scientific, Cat. # TS-20684), 0.4 μL of 10 mM dNTP (Promega, Cat. # U1511), 0.2 μL of 10 μM forward primer, 0.2 μL of 10 μM reverse primer, 0.2 μL of template DNA, and 0.1 μL of Phusion High-Fidelity DNA Polymerase (Thermo Scientific, Cat. # F-530S). When needed, water was substituted with 20% acetamide. The reactions were performed in a Mastercycler EP Gradient (Eppendorf) with the following cycle: a DNA melting step at 94°C for 22 s, an annealing step at 58.1°C for 35 s, and an extension step at 72°C for 90 s. This cycle was repeated 40 times, after which a final annealing step at 72°C for 5 min was performed. Template DNA included *Hfx. volcanii* DS2 genomic DNA (20 ng/μL), plasmid DNA listed in Table 1, and DNA from *E. coli* and *Hfx. volcanii* colonies.

**Table 2:** **List of primers used in this study**.

**Primer name**	**Description**	**Sequence**
dhaKLM_FR1_F	Used to amplify flanking regions of *dhaKLM* for insertion into pTA131 digested with HindIII and BamHI to delete the operon.	5′- CGG TAT CGA TAA GCT GCC CTA CGC ACC CTA CAT G -3′
dhaKLM_FR1_R		5′- TAG AAC TAG TGG ATC GCC TTC GGC TAC CCG CTC AT -3′
dhaKLM_FR2_F		5′- GGA ATT CTA CCA GGC TCT GCG CTG AAC CGG CCG AA -3′
dhaKLM_FR2_R		5′- GCC TGG TAG AAT TCC GAC TCA CCG TCC CTC ACG TT -3′
dhaKLMF	Used to amplify *dhaKLM* and native promoter for insertion into pTA409 digested with BamHI and XhoI to complement the operon.	5′- TAG AAC TAG TGG ATC AGG CGG TCG CGC GTT TCC GT -3′
dhaKLMR		5′- CGG GCC CCC CCT CGA ATC AGT TCA GCT TCC GGT AGT CGC G -3′
glpK_FR1F	Used to amplify flanking regions of *glpK* for insertion into pTA131 digested with XhoI and XbaI to delete gene [external primers based on designs from Sherwood et al. (2009)].	5′- CGG GCC CCC CCT CGA TCG ACG ACC AGG CGT -3′
glpK_FR1R		5′- TGG CGG CCG CTC TAG ACG ATG ACA ACG ATG T -3′
glpK_FR2F		5′- GCC TGG GCA GAT CTC AAC ACG TGT TCG AAG -3′
glpK_FR2R		5′- GAG ATC TGC CCA GGC TTC TAA CCA ACC TCG ATA CG -3′
glpKF	Used to amplify glpK and native promoter for insertion into pTA409 digested with BamHI and XhoI to complement gene.	5′- CGG GCC CCC CCT CGA CGC ACA ACT GAC GAA CGG GA -3′
glpKR		5′- TAG AAC TAG TGG ATC TTA TTC CTC CCG TGC CCA GTC -3′

Gel electrophoresis was performed to separate and analyze the PCR products using 0.8% (w/v) agarose in 1 × TAE buffer (40 mM Tris acetate, 2 mM EDTA). After gel electrophoresis, PCR products were excised from the gel and purified using the Wizard SV Gel and PCR Clean-Up System (Promega). Plasmids from *E. coli* strains were extracted and purified using the PureYield Plasmid Miniprep System (Promega). Plasmids linearized via digestion with restriction enzymes (BamHI, HindIII, XhoI, or XbaI) were also purified using the Wizard SV Gel and PCR Clean-Up System.

### Gene deletion in *Hfx. volanii*

**Figure 1:**
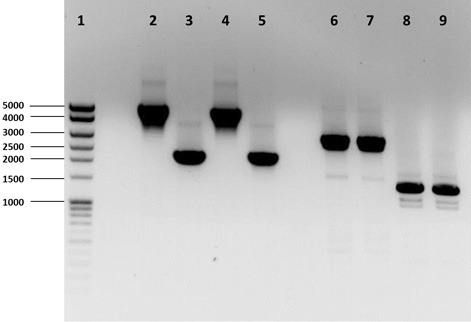
**PCR analysis of H26, Δ*dhaKLM*, Δ*glpK*, and Δ*dhaKLM* Δ*glpK***. Analysis examined presence or absence of the *dhaKLM* operon (lanes 2–5) and *glpK* (lanes 6–9). Lane 1 contained exACTGene Mid Range Plus DNA Ladder (Fisher Scientific). Lanes 2 and 6 contained amplicons from H26. Lanes 3 and 7 contained amplicons from Δ*dhaKLM*. Lanes 4 and 8 contained amplicons from Δ*glpK*. Lanes 5 and 9 contained amplicons from Δ*dhaKLM* Δ*glpK*.

Three *Hfx. volcanii* genes (*dhaKLM*; HVO_1544, HVO_1545, and HVO_1546), which encode homologs to the putative DHA kinase genes in *Hqm. walsbyi*, and a glycerol kinase gene (*glpK*; HVO_1541), were targeted for deletion in *Hfx. volcanii* strain H26 using the In-Fusion HD Cloning Kit (Clontech). The strategy for gene deletion was based on the methodology outlined in a study by Blaby et al. (2010) with a few modifications. Flanking regions of the targeted genes were developed to be between 800 and 1000 bp in length. The 15-bp linker used to combine the flanking regions was altered to so that EcoRI and BstOI sites were included for the *dhaKLM* deletion linker and BglI and BstOI sites were included for the *glpK* deletion linker. The pTA131 was linearized with HindIII and BamHI for the *dhaKLM* deletion and XhoI and XbaI for the *glpK* deletion. Constructed plasmids were transformed into Stellar Competent Cells (Clontech, Cat. # 636763), according to the directions of the provider, and were plated on LB-amp plates with X-gal. White colonies were screened via colony PCR using the external primers of the target gene flanking regions. Confirmed deletion plasmids (listed in Table 1) were subcloned in *dam^−^/dcm^−^* Competent *E. coli* (New England BioLabs, Cat. # C2925H) to produce demethylated plasmids for transformation of *Hfx. volcanii*. *Hfx. volcanii* H26 colonies were screened for deleted genes via PCR using the external primers of the target gene flanking regions. The size of PCR products of screened cells were compared to those produced with wild-type DNA (Figure 1). Smaller product size indicated that the gene had been deleted. The *Hfx. volcanii* H26 deletion strains produced by this process are listed in Table 1.

### Complementation of deleted genes

The *dhaKLM* and *glpK* genes deleted in *Hfx. volcanii* H26 were resuscitated by constructing complementation plasmids. Primers were designed which amplified the upstream native promoter and the coding region of the targeted genes in *Hfx. volcanii*. The primers were also designed to have 15 bp of homology with pTA409. Restriction digestion of pTA409 was performed using BamHI and XhoI to linearize the plasmid. After the linearized pTA409 and gene fragments were gel-purified, the DNA fragments were combined together using the In-Fusion HD Cloning Kit according to the instructions of the provider. The constructed plasmids were cloned, screened, and demethylated as described in the above gene deletion protocol. Purified constructed plasmids (listed in Table 1) were then transformed into the *Hfx. volcanii* H26 deletion strains using the PEG mediated transformation of Haloarchaea protocol from *The Halohandbook*. PCR was used to confirm transformation success. The *Hfx. volcanii* complementation strains produced by this process are listed in Table 1.

### DHA growth experiments

*Hfx. volcanii* strains listed in Table 1 were grown to late-exponential phase (OD_600_ = ~ 0.6−0.8) in Hv-YPC medium. The cell cultures were then centrifuged at 3220 RCF for 15 min and resuspended in Hv-min -C media supplemented with uracil. Centrifugation was repeated a total of three times to wash the cells of residual Hv-YPC media. During the final resuspension of the cells in Hv-min -C media, the cell cultures were diluted to OD_600_ ~0.01. Each cell culture was then distributed into the wells of a 96-well plate, with each well receiving 190 μL of cell culture. Also, 200 μL of Hv-min -C was added to the plate to be used as a blank. Three wells of each culture were treated with 10 μL of either 0.1 M DHA (final concentration of 5 mM DHA), 0.05 M DHA (final concentration of 2.5 mM DHA), 0.02 M DHA (final concentration of 1 mM DHA), or deionized water (negative control). The 96-well plate was then placed into a Multiscan FC plate reader (Fisher Scientific), which incubated the plate at 42°C while shaking it at low speed. The plate reader measured the OD_620_ of each well every hour for 72 h.

### Bioinformatics

The amino acid sequences of the *Hfx. volcanii* putative DHA kinase gene *dhaK* (HVO_1546) and glycerol kinase gene *glpK* were used to perform BLASTp (http://blast.ncbi.nlm.nih.gov/Blast.cgi) searches of the NCBI database to determine other halobacterial species with DHA kinase and glycerol kinase genes. The amino acid sequences were retrieved from the NCBI database (*dhaK* GI number 292655696; *glpK* GI number 292655691). The search was restricted to the Halobacteriales (taxid 2235) with an *E*-value cut-off of 1e-20. Reciprocal BLASTp was performed to analyze only orthologous genes. The halobacterial genomes queried in this BLASTp search are listed in Table 3.

**Table 3:** **List of halobacterial genomes queried in BLASTp search**.

**Queried halobacterial genomes**			
*Haladaptatus paucihalophilus DX253*	*Haloferax larsenii JCM 13917*	*Halorubrum aidingense JCM 13560*	*Natrialba asiatica DSM 12278*
*Halalkalicoccus jeotgali B3*	*Haloferax lucentense DSM 14919*	*Halorubrum tebenquichense DSM 14210*	*Natrialba chahannaoensis JCM 10990*
*Halarchaeum acidiphilum MH1-52-1*	*Haloferax denitrificans ATCC 35960*	*Halorubrum terrestre JCM 10247*	*Natrialba hulunbeirensis JCM 10989*
*Haloarcula amylolytica JCM 13557*	*Haloferax elongans ATCC BAA-1513*	*Halorubrum arcis JCM 13916*	*Natrialba magadii ATCC 43099*
*Haloarcula argentinensis DSM 12282*	*Haloferax gibbonsii ATCC 33959*	*Halorubrum californiensis DSM 19288*	*Natrialba taiwanensis DSM 12281*
*Haloarcula californiae ATCC 33799*	*Haloferax mediterranei ATCC 33500*	*Halorubrum coriense DSM 10284*	*Natrinema altunense JCM 12890*
*Haloarcula hispanica ATCC 33960*	*Haloferax mucosum ATCC BAA-1512*	*Halorubrum distributum JCM 9100*	*Natrinema gari JCM 14663*
*Haloarcula japonica DSM 6131*	*Haloferax prahovense DSM 18310*	*Halorubrum ezzemoulense DSM 17463*	*Natrinema pallidum DSM 3751*
*Haloarcula marismortui ATCC 43049*	*Haloferax sulfurifontis ATCC BAA-897*	*Halorubrum hochstenium ATCC 700873*	*Natrinema pellirubrum DSM 15624*
*Haloarcula sinaiiensis ATCC 33800*	*Haloferax volcanii DS2*	*Halorubrum kocurii JCM 14978*	*Natrinema versiforme JCM 10478*
*Haloarcula vallismortis ATCC 29715*	*Haloferax* sp. *ATCC BAA-644*	*Halorubrum lacusprofundi ATCC 49239*	*Natrinema* sp. *CX2021*
*Haloarcula* sp. *AS7094*	*Haloferax* sp. *ATCC BAA-645*	*Halorubrum lipolyticum DSM 21995*	*Natrinema* sp. *J7-1*
*Halobacterium salinarum NRC-1*	*Haloferax* sp. *ATCC BAA-646*	*Halorubrum litoreum JCM 13561*	*Natrinema* sp. *J7-2*
*Halobacterium* sp. *DL1*	*Haloferax* sp. *BAB2207*	*Halorubrum saccharovorum DSM 1137*	*Natronobacterium gregoryi SP2*
*Halobacterium* sp. *GN101*	*Halogeometricum borinquense DSM 11551*	*Halorubrum* sp. *T3*	*Natronobacterium* sp. *AS-7091*
*Halobaculum gomorrense JCM 9908*	*Halogranum salarium B-1*	*Halosarcina pallida JCM 14848*	*Natronococcus amylolyticus DSM 10524*
*Halobiforma lacisalsi AJ5*	*Halomicrobium katesii DSM 19301*	*Halosimplex carlsbadense 2-9-1*	*Natronococcus jeotgali DSM 18795*
*Halobiforma nitratireducens JCM 10879*	*Halomicrobium mukohataei DSM 12286*	*Halostagnicola larsenii XH-48*	*Natronococcus occultus SP4*
*Halococcus hamelinensis 100A6*	*Halopiger xanaduensis SH-6*	*Haloterrigena limicola JCM 13563*	*Natronolimnobius innermongolicus JCM 12255*
*Halococcus morrhuae DSM 1307*	*Halopiger* sp. *IIH2*	*Haloterrigena salina JCM 13891*	*Natronomonas moolapensis 8.8.11*
*Halococcus saccharolyticus DSM 5350*	*Halopiger* sp. *IIH3*	*Haloterrigena thermotolerans DSM 11522*	*Natronomonas pharaonis DSM 2160*
*Halococcus salifodinae DSM 8989*	*Haloplanus natans DSM 1798*	*Haloterrigena turkmenica DSM 5511*	*Natronorubrum bangense JCM 10635*
*Halococcus thailandensis JCM 13552*	*Haloquadratum walsbyi DSM 16790*	*Halovivax asiaticus JCM 14624*	*Natronorubrum sulfidifaciens JCM 14089*
*Halococcus* sp. *197A*	*Halorhabdus tiamatea SARL4B*	*Halovivax ruber XH-70*	*Natronorubrum tibetense GA33*
*Haloferax alexandrinus JCM 10717*	*Halorhabdus utahensis DSM 12940*	*Natrialba aegyptia DSM 13077*	*Salinarchaeum* sp. *Harcht-Bsk1*

## Results

### DHA kinase is patchily distributed among the halobacteria

Three DHA kinase genes (HQ2672A, HQ2673A, and HQ2674A) have been annotated in the genome of *Hqm. walsbyi* (Bolhuis et al., 2006), a halobacterial species which is able to metabolize external DHA (Elevi Bardavid and Oren, 2008). Homologs of these three genes are also annotated in *Hfx. volcanii* (HVO_1544, HVO_1545, and HVO_1546). In order to determine the prevalence of DHA kinase genes among the Halobacteria, the *Hfx. volcanii dhaK* gene (HVO_1546) was used to perform a BLASTp search against the database of Halobacteria genomes available on NCBI. The search yielded significant hits among 31 different halobacterial species (Table 4). Except for *Haloferax larsenii* and *Haloferax elongans*, all queried *Haloferax* species yielded significant hits in the BLASTp search. Species from the *Halobiforma, Halococcus, Halorubrum*, and *Natronococcus* genera also yielded significant hits, but not all queried species from these genera produced results. All representatives from the genera *Haladaptatus, Halalkalicoccus, Halarchaeum, Haloquadratum, Halosarcina*, and *Salinarchaeum* yielded significant hits. Halobacteria genera that did not yield significant hits in the BLASTp search (*E*-value cut-off of 1e-20) include *Haloarcula, Halobacterium, Halobaculum, Halogeometricum, Halogranum, Halomicrobium, Halopiger, Haloplanus, Halorhabdus, Halosimplex, Halostagnicola, Haloterrigena, Halovivax, Natrialba, Natrinema, Natronobacterium, Natronolimnobius, Natronomonas*, and *Natronorubrum*.

**Table 4:** **Results of BLASTp search using *dhaK* (Performed on July 29, 2013)**.

**Species name**	**GI number**	***E*-value**	**Species name**	**GI number**	***E*-value**
*Haloferax volcanii DS2*	292655696	0.0	*Natronococcus amylolyticus DSM 10524*	491710546	1e-169
*Haloferax* sp. *BAB2207*	493648700	0.0	*Halarchaeum acidiphilum MH1-52-1*	519064717	2e-169
*Haloferax alexandrinus JCM 10717*	445742333	0.0	*Halogranum salarium B-1*	496767283	3e-165
*Haloferax sulfurifontis ATCC BAA-897*	494484188	0.0	*Halorubrum lipolyticum DSM 21995*	495278338	7e-165
*Haloferax lucentense DSM 14919*	490164612	0.0	*Halorubrum* sp. *T3*	515912844	2e-164
*Haloferax denitrificans ATCC 35960*	491112269	0.0	*Halorubrum kocurii JCM 14978*	496125287	3e-164
*Haloferax mediterranei ATCC 33500*	389847061	0.0	*Halococcus hamelinensis 100A6*	494968649	2e-162
*Haloferax* sp. *ATCC BAA-644*	445718309	0.0	*Halosarcina pallida JCM 14848*	495659148	2e-160
*Haloferax* sp. *ATCC BAA-645*	445712370	0.0	*Halorubrum lacusprofundi ATCC 49239*	222479879	6e-158
*Haloferax* sp. *ATCC BAA-646*	495849737	0.0	*Halococcus saccharolyticus DSM 5350*	492981238	3e-157
*Haloferax gibbonsii ATCC 33959*	491118466	0.0	*Halorubrum aidingense JCM 13560*	495274943	3e-157
*Haloferax prahovense DSM 18310*	445719493	0.0	*Haloquadratum walsbyi DSM 16790*	110668578	2e-154
*Haloferax mucosum ATCC BAA-1512*	495592772	0.0	*Halorubrum coriense DSM 10284*	493055434	6e-154
*Haladaptatus paucihalophilus DX253*	495255891	0.0	*Salinarchaeum* sp. *Harcht-Bsk1*	495690630	2e-150
*Halobiforma lacisalsi AJ5*	494236904	9e-180	*Halalkalicoccus jeotgali B3*	300710867	3e-145
*Natronococcus jeotgali DSM 18795*	495699224	2e-178			

### Growth on DHA in *Hfx. volcanii* is concentration dependent

**Figure 2:**
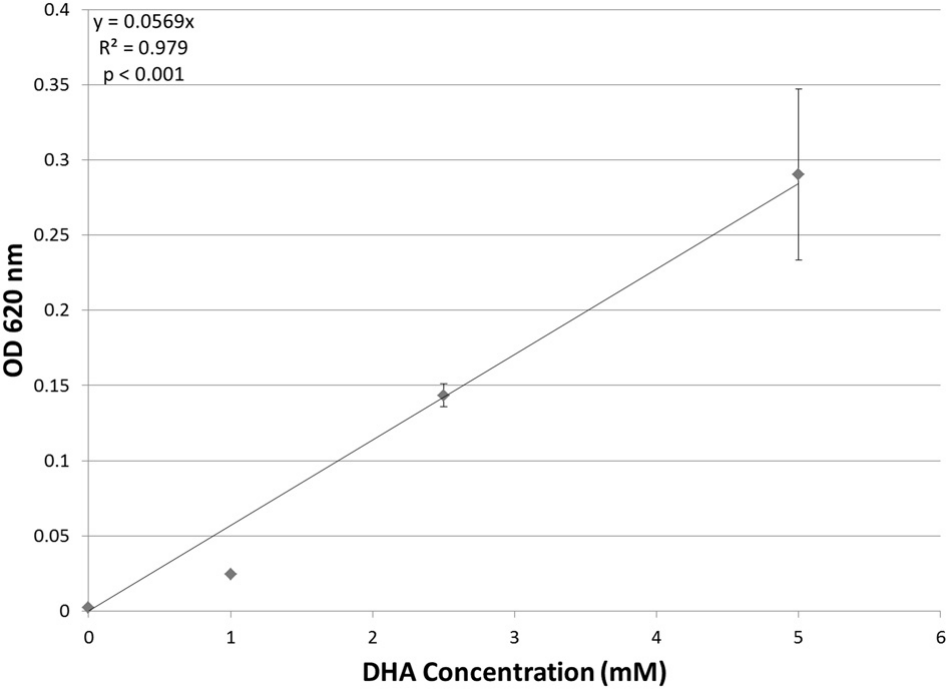
**Cell density of *H. volcanii* H26 at stationary phase vs. concentration of DHA**. Cell density is represented by the average optical density (OD_620_) reading of three cell culture replicates. Error bars depict the standard deviation of the averages. The depicted line represents the line of best fit for the data. ANOVA single factor, *p* < 0.001.

Although putative DHA kinase genes are present in *Hfx. volcanii*, no previous research has demonstrated that *Hfx. volcanii* is able to grow on DHA as a carbon source. Therefore, experiments were performed to test the growth of *Hfx. volcanii* strain H26 on 5 mM, 2.5 mM, and 1 mM DHA. The results indicated that H26 was capable of growth on DHA as the sole carbon source. The cell density at which H26 reached stationary phase was also dependent on the initial concentration of DHA provided to the cells (Figure 2). H26 cells grown in medium supplemented with 1 mM DHA reached stationary phase at the lowest cell density, whereas cells grown with the highest tested concentration of 5 mM DHA reached stationary phase at the highest cell density. These data indicate that growth of *Hfx. volcanii* on DHA as a carbon source is concentration dependent.

### DHA kinase is used in DHA metabolism in *Hfx. volcanii*

**Figure 3:**
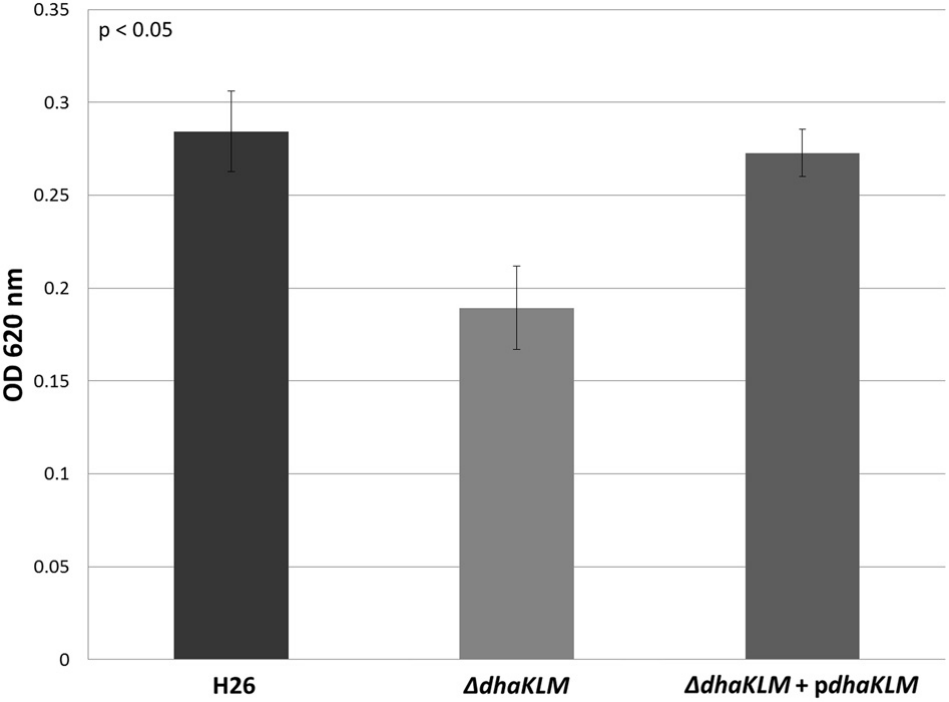
**Cell density of H26, Δ*dhaKLM*, and Δ*dhaKLM +* p*dhaKLM* at stationary phase when grown on 5 mM DHA**. Cell density is represented by the average optical density (OD_620_) reading of three cell culture replicates. Error bars depict the standard deviation of the averages. ANOVA single factor, *p* < 0.05.

Evidence indicates that *Hfx. volcanii*, like *Hqm. walsbyi*, can use DHA as a carbon source. Although both organisms have DHA kinase genes, no previous studies demonstrated these putative DHA kinase genes have a role in DHA metabolism. In order to determine that DHA metabolism in *Hfx. volcanii* utilizes the annotated DHA kinase, the operon *dhaKLM* (HVO_1544—HVO_1546) was deleted in *Hfx. volcanii* strain H26. The growth of this deletion strain (Δ*dhaKLM*) on 5 mM DHA was then tested in comparison to the parent strain H26 as well as a complementation strain (Δ*dhaKLM* + p*dhaKLM*). The results indicate that the deletion of *dhaKLM* causes a reduction in growth on DHA, and that complementation of the deleted genes negates this growth deficiency (Figure 3). However, the Δ*dhaKLM* was still capable of growth on DHA, exhibiting a 33% decrease in growth compared to H26. These results indicate that the *dhaKLM* genes are used by *Hfx. volcanii* in DHA metabolism, most likely for the phosphorylation of DHA to DHA phosphate, and that the genes are apparently not essential. Since it is still capable of growth on DHA there must be additional genes involved in the phosphorylation step.

### Glycerol kinase is more important than DHA kinase

**Figure 4:**
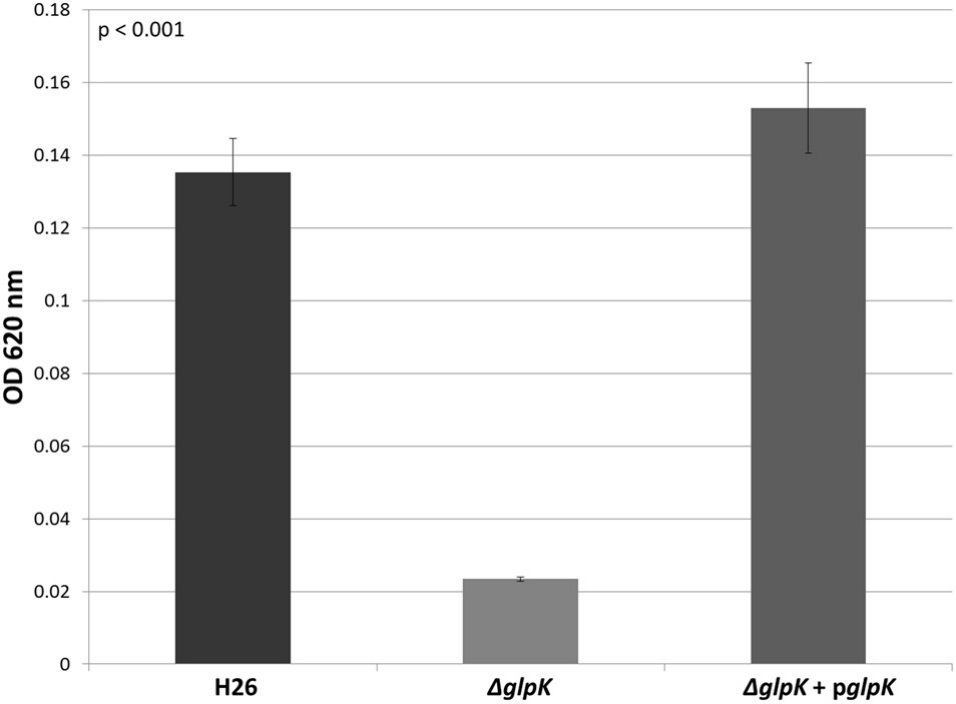
**Cell density of H26, Δ*glpK*, and Δ*glpK +* p*glpK* at stationary phase when grown on 5 mM DHA**. Cell density is represented by the average optical density (OD_620_) reading of three cell culture replicates. Error bars depict the standard deviation of the averages. ANOVA single factor, *p* < 0.001.

In other organisms, glycerol kinase is also capable of phosphorylating DHA (Hayashi and Lin, 1967; Weinhouse and Benziman, 1976; Jin et al., 1982). Therefore, the other gene involved DHA metabolism in *Hfx. volcanii* was hypothesized to be the glycerol kinase gene *glpK* (HVO_1542). In order to test this hypothesis, the *glpK* gene was deleted in H26. The deletion strain (Δ*glpK*), and its complementation strain (Δ*glpK* + p*glpK*), were both grown on 5 mM DHA along with the parent strain H26. The results indicate that the deletion of *glpK* caused a reduction in growth on DHA even greater than deletion of *dhaKLM*, and that complementation of the *glpK* gene restores growth to normal levels (Figure 4). In comparison to the parent strain H26, Δ*glpK* strain demonstrated an 83% decrease in growth. This decrease is far greater than the 33% decrease exhibited by the Δ*dhaKLM* deletion mutant. These results indicate that the *glpK* gene is used by *Hfx. volcanii* in DHA metabolism, and that its role is potentially greater than that of the *dhaKLM* operon.

**Figure 5:**
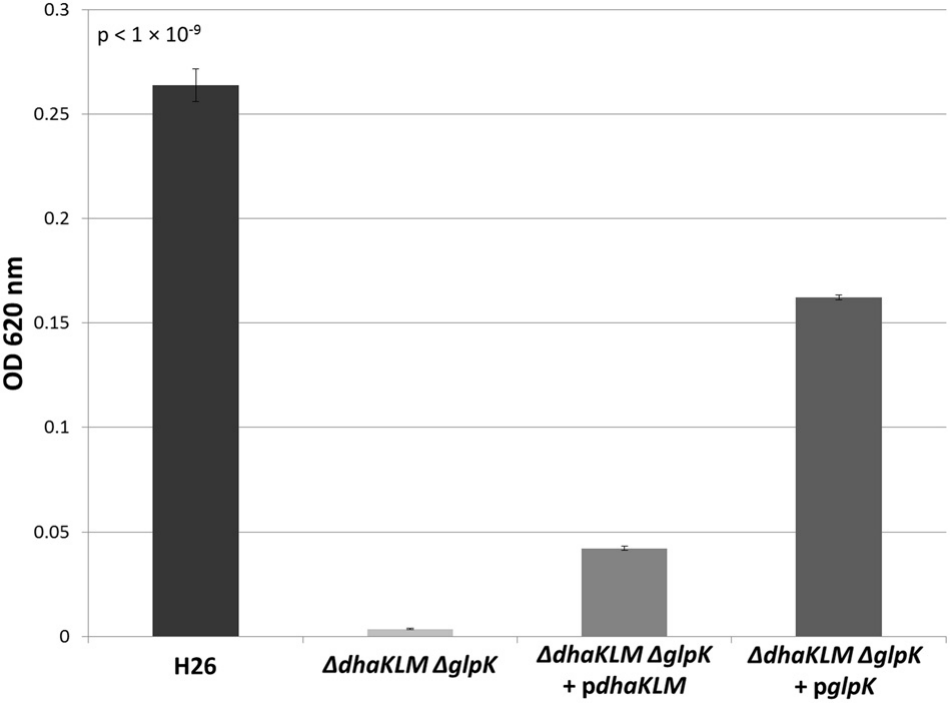
**Cell density of H26, Δ*dhaKLM* Δ*glpK*, Δ*dhaKLM* Δ*glpK* + p*dhaKLM*, and Δ*dhaKLM* Δ*glpK* + p*glpK* at stationary phase when grown on 5 mM DHA**. Cell density is represented by the average optical density (OD_620_) reading of three cell culture replicates. Error bars depict the standard deviation of the averages. ANOVA single factor, *p* < 1 × 10^−9^.

In order to further test the roles of the DHA kinase and glycerol kinase in DHA metabolism in *Hfx. volcanii*, the *dhaKLM* operon and *glpK* gene were both deleted in H26. This double deletion mutant (Δ*dhaKLM ΔglpK*), along with a DHA kinase complementation strain (Δ*dhaKLM ΔglpK* + p*dhaKLM*), a glycerol kinase complementation strain (Δ*dhaKLM ΔglpK* + p*glpK*), and the parent strain H26, were then grown on 5 mM DHA. The results indicate that the deletion of both kinases abolishes growth on DHA, and that complementation with glycerol kinase restores growth to a greater degree than complementation with DHA kinase (Figure 5). The Δ*dhaKLM ΔglpK* strain did not exhibit any growth, remaining at the initial OD_620_ of 0.0035. The Δ*dhaKLM ΔglpK* + p*dhaKLM* strain was able to grow on DHA, but demonstrated an 84% decrease compared to the H26 parent strain. The Δ*dhaKLM ΔglpK* + p*glpK* was also capable of limited growth on DHA, but demonstrated a 39% growth decrease from H26 and a 390% growth increase compared with Δ*dhaKLM ΔglpK* + p*dhaKLM*. Overall, these data confirm that glycerol kinase is more important for DHA metabolism in *Hfx. volcanii* than DHA kinase.

### Glycerol kinase is widely distributed among the halobacteria

Since growth experiments indicated that glycerol kinase has a significant role in DHA metabolism, the presence of this gene in halobacterial species could potentially be a determinant of DHA metabolism in those species. Although the distribution of *glpK* homologs has been examined in previous studies (Sherwood et al., 2009; Anderson et al., 2011), a greater number of halobacterial genomes have become available since those studies. Therefore, the *glpK* gene in *Hfx. volcanii* was used to perform a BLASTp search against the halobacterial genomes available on NCBI. The search yielded 90 significant hits among 82 different species of Halobacteria (Table 5), indicating a much wider distribution of glycerol kinase compared to DHA kinase among the Halobacteria. Six species yielded more than one significant hit: *Halogeometricum borinquense* (3 hits), *Haladaptatus paucihalophilus* (3 hits), *Haloferax prahovense* (2 hits), *Haloferax mucosum* (2 hits), *Haloferax gibbonsii* (2 hits), and *Natronomonas moolapensis* (2 hits). The multiple hits indicate the presence of *glpK* paralogs in these species. Only 18 of the 100 queried halobacterial species did not yield significant hits: *Haloarcula* sp. *AS7094, Halobacterium* sp. *DL1, Halobacterium* sp. *GN101, Halobaculum gomorrense, Halococcus* sp. *197A, Halopiger* sp. *IIH2, Halopiger* sp. *IIH3, Haloplanus natans, Halorubrum ezzemoulense, Halosarcina pallida, Halostagnicola larsenii, Halovivax asiaticus, Halovivax ruber, Natrinema* sp. *CX2021, Natrinema* sp. *J7-1, Natronobacterium gregoryi, Natronobacterium* sp. *AS-7091*, and *Natronomonas pharaonis*. It should be noted, however, that only the genomes of *Halovivax ruber, Natronobacterium gregoryi*, and *Natronomonas pharaonis* are completely sequenced, whereas the other genomes without significant hits are incomplete, leaving open the possibility that these species might have *glpK* homologs. With the exception of *Halosarcina pallida*, which has an incompletely sequenced genome, all halobacterial species that yielded significant hits in the *dhaK* BLASTp search also yielded significant hits in the *glpK* BLASTp search.

**Table 5:** **Results of BLASTp search using *glpK* (Performed on September 17, 2013)**.

**Species name**	**GI number**	***E*-value**	**Species name**	**GI number**	***E*-value**
*Haloferax volcanii DS2*	292655691	0.0	*Haloferax mucosum ATCC BAA-1512*	445745541	0.0
*Haloferax* sp. *BAB2207*	432200129	0.0	*Natrialba hulunbeirensis JCM 10989*	445640226	0.0
*Haloferax lucentense DSM 14919*	445722906	0.0	*Halarchaeum acidiphilum MH1-52-1*	543417579	0.0
*Haloferax alexandrinus JCM 10717*	445742338	0.0	*Halorubrum californiensis DSM 19288*	445688091	0.0
*Haloferax* sp. *ATCC BAA-646*	445709004	0.0	*Halorubrum lipolyticum DSM 21995*	445813038	0.0
*Haloferax* sp. *ATCC BAA-645*	445712375	0.0	*Halorubrum lacusprofundi ATCC 49239*	222479549	0.0
*Haloferax* sp. *ATCC BAA-644*	445718304	0.0	*Salinarchaeum* sp. *Harcht-Bsk1*	510882182	0.0
*Haloferax sulfurifontis ATCC BAA-897*	445746251	0.0	*Natrialba chahannaoensis JCM 10990*	445643664	0.0
*Haloferax denitrificans ATCC 35960*	445749875	0.0	*Halorubrum hochstenium ATCC 700873*	445701406	0.0
*Haloferax prahovense DSM 18310*	445719488	0.0	*Halomicrobium mukohataei DSM 12286*	257388556	0.0
*Haloferax elongans ATCC BAA-1513*	445734605	0.0	*Halorubrum tebenquichense DSM 14210*	445687222	0.0
*Haloferax larsenii JCM 13917*	445729767	0.0	*Haloarcula amylolytica JCM 13557*	445772086	0.0
*Haloferax gibbonsii ATCC 33959*	445726194	0.0	*Halomicrobium katesii DSM 19301*	517069632	0.0
*Haloferax mediterranei ATCC 33500*	389847056	0.0	*Halosimplex carlsbadense 2-9-1*	445671661	0.0
*Haloferax mucosum ATCC BAA-1512*	445747425	0.0	*Haloarcula vallismortis ATCC 29715*	445755712	0.0
*Halogeometricum borinquense DSM 11551*	313125210	0.0	*Haloarcula argentinensis DSM 12282*	445773756	0.0
*Halogeometricum borinquense DSM 11551*	313126426	0.0	*Halorubrum litoreum JCM 13561*	445813470	0.0
*Halobiforma nitratireducens JCM 10879*	445784518	0.0	*Haloarcula marismortui ATCC 43049*	55377424	0.0
*Natrinema pallidum DSM 3751*	445622526	0.0	*Haloarcula sinaiiensis ATCC 33800*	445762583	0.0
*Haladaptatus paucihalophilus DX253*	320548735	0.0	*Haloarcula californiae ATCC 33799*	445763060	0.0
*Haloterrigena salina JCM 13891*	445666802	0.0	*Natronolimnobius innermongolicus JCM 12255*	445597617	0.0
*Haloterrigena thermotolerans DSM 11522*	445659630	0.0	*Natronorubrum tibetense GA33*	445585740	0.0
*Natrinema pellirubrum DSM 15624*	433590333	0.0	*Haloarcula japonica DSM 6131*	445778554	0.0
*Halococcus morrhuae DSM 1307*	445795889	0.0	*Natrialba aegyptia DSM 13077*	445651647	0.0
*Haloterrigena limicola JCM 13563*	445665007	0.0	*Natrialba taiwanensis DSM 12281*	445642534	0.0
*Halococcus salifodinae DSM 8989*	445798601	0.0	*Haloquadratum walsbyi DSM 16790*	110667688	0.0
*Halococcus hamelinensis 100A6*	445790305	0.0	*Natrialba magadii ATCC 43099*	289580614	0.0
*Natrinema* sp. *J7-2*	397773488	0.0	*Natrialba asiatica DSM 12278*	445650101	0.0
*Natrinema altunense JCM 12890*	445633695	0.0	*Natronomonas moolapensis 8.8.11*	452208319	0.0
*Natronorubrum sulfidifaciens JCM 14089*	445594250	0.0	*Haloferax gibbonsii ATCC 33959*	445728401	0.0
*Natrinema gari JCM 14663*	445628815	0.0	*Natronococcus jeotgali DSM 18795*	445603927	0.0
*Halococcus thailandensis JCM 13552*	445801492	0.0	*Halorubrum saccharovorum DSM 1137*	445683831	0.0
*Natrinema versiforme JCM 10478*	445613765	0.0	*Halorhabdus utahensis DSM 12940*	257052548	0.0
*Halobiforma lacisalsi AJ5*	445778236	0.0	*Halogranum salarium B-1*	399240308	0.0
*Haladaptatus paucihalophilus DX253*	320549923	0.0	*Halorubrum arcis JCM 13916*	445822264	0.0
*Haloterrigena turkmenica DSM 5511*	284166225	0.0	*Halorubrum terrestre JCM 10247*	445683460	0.0
*Haladaptatus paucihalophilus DX253*	516847391	0.0	*Halorubrum distributum JCM 9100*	445698917	0.0
*Halalkalicoccus jeotgali B3*	300711495	0.0	*Haloarcula hispanica ATCC 33960*	344211542	0.0
*Natronococcus occultus SP4*	435847946	0.0	*Halorubrum kocurii JCM 14978*	445806839	0.0
*Halopiger xanaduensis SH-6*	336253699	0.0	*Halorhabdus tiamatea SARL4B*	529078002	0.0
*Natronococcus amylolyticus DSM 10524*	445599450	0.0	*Halorubrum* sp. *T3*	515912305	0.0
*Halogeometricum borinquense DSM 11551*	445572938	0.0	*Halorubrum aidingense JCM 13560*	445818937	0.0
*Natronorubrum bangense JCM 10635*	445597786	0.0	*Halorubrum coriense DSM 10284*	445694991	0.0
*Haloferax prahovense DSM 18310*	445713901	0.0	*Natronomonas moolapensis 8.8.11*	452206238	0.0
*Halococcus saccharolyticus DSM 5350*	445793423	0.0	*Halobacterium salinarum NRC-1*	15790841	0.0

## Discussion

Previously, *Hqm. walsbyi* was the only halobacterial species known to be able to utilize DHA as a carbon source (Elevi Bardavid and Oren, 2008). In this study, we have identified *Hfx. volcanii* as the second halobacterial species known to be capable of metabolizing DHA. When DHA was added to growth medium as the sole carbon source, *Hfx. volcanii* was capable of growth. This growth was variable based on the concentration of DHA present in the growth medium. The ability of *Hfx. volcanii* to metabolize DHA suggests that the substrate could be an important carbon source in the Dead Sea environment where *Hfx. volcanii* naturally lives. Elevi Bardavid and Oren (2008) have suggested that *Salinibacter* might be a source of DHA in hypersaline environments, since it can produce DHA as an overflow product. However, *Salinibacter* has not been identified in the Dead Sea, making it an unlikely candidate for DHA producer. The DHA could potentially be produced as an overflow product from *Dunaliella parva*, a halophilic alga that is the most prominent photosynthetic organism in the Dead Sea and is able to produce DHA (Ben-Amotz and Avron, 1974; Oren and Shilo, 1982). Elevi Bardavid and Oren (2008) hypothesized that the *Dunaliella* cell membrane could be permeable to DHA, allowing excess DHA produced by the cells to leak into the external environment. If *D. parva* produces a significant amount of DHA overflow, the substrate would be readily available for *Hfx. volcanii* to utilize as a source of carbon.

When Elevi Bardavid and Oren (2008) demonstrated that *Hqm. walsbyi* could utilize DHA as a carbon source, they hypothesized that the organism used a system involving a PEP-dependent DHA kinase to phosphorylate DHA to DHA kinase, based on genomic analysis from Bolhuis et al. (2006). However, their study did not demonstrate a direct connection between the putative DHA kinase and DHA metabolism. In our model halobacterial organism, *Hfx. volcanii*, we have demonstrated that DHA kinase is involved in metabolism of DHA. When the DHA kinase operon *dhaKLM* is deleted, growth of *Hfx. volcanii* on DHA is impeded, and complementation of the deleted genes with the *dhaKLM* operon restores growth. The growth of *Hfx. volcanii* is not completely abolished, however, and further analysis using a strain wherein the glycerol kinase gene *glpK* has been deleted indicates that *Hfx. volcanii* also uses glycerol kinase for DHA metabolism. Deletion of the *glpK* gene reduces growth on DHA more dramatically than the *dhaKLM* deletion, indicating that the role of glycerol kinase is more pronounced in DHA metabolism than that of DHA kinase for *Hfx. volcanii*. This enzyme primacy is further supported by the observation that, in the double deletion mutant Δ*dhaKLM ΔglpK*, complementation with *glpK* restores growth better than complementation with *dhaKLM*.

The primacy of the glycerol kinase in DHA metabolism is unexpected, since DHA kinase is usually the primary enzyme involved in DHA phosphorylation in other organisms due to the lower affinity of glycerol kinase for DHA. In *Klebsiella pneumoniae*, the glycerol kinase has a *K*_*m*_ of 1 × 10^−3^ M for DHA, whereas the DHA kinase has a *K*_*m*_ of 1 × 10^−5^ M (Jin et al., 1982). The glycerol kinase in *E. coli* has a *K*_*m*_ of 5 × 10^−4^ M for DHA (Hayashi and Lin, 1967), but the DHA kinase has a *K*_*m*_ of 4.5 × 10^−7^ M (Gutknecht et al., 2001). One possible explanation for the primacy of the glycerol kinase in *Hfx. volcanii* DHA metabolism is the glycerol kinase might have a higher affinity than DHA kinase for DHA. Another possible explanation might be differences in expression of the kinases. DHA kinase might be expressed at lower levels than glycerol kinase early in the *Hfx. volcanii* growth cycle, which would cause the glycerol kinase to be the primary DHA phosphorylating enzyme despite a possible lower affinity for DHA. Later in the growth cycle, however, *Hfx. volcanii* may increase expression of DHA kinase, leading to the higher affinity enzyme becoming the new primary enzyme for DHA phosphorylation. Growth experiments of Δ*dhaKLM ΔglpK +* p*dhaKLM*, in which the strain was grown beyond 72 h on 5 mM DHA, support this hypothesis, since growth of the strain on DHA increased significantly after 80 h, and actually surpassed Δ*dhaKLM ΔglpK +* p*glpK* after 96 h (data not shown). In-depth analysis into the enzymatic activity and kinetic constants of these enzymes toward DHA, as well as their expression levels, would enhance understanding on glycerol kinase primacy in *Hfx. volcanii* DHA metabolism.

DHA metabolism among the Halobacteria may extend beyond *Hfx. volcanii* and *Hqm. walsbyi*. Our BLASTp results for *dhaK* indicate that 29 other halobacterial species have a DHA kinase gene homologous to *dhaK* in *Hfx. volcanii* and *Hqm. walsbyi*. Since our data indicate that the *dhaKLM* genes in *Hfx. volcanii* are involved in DHA metabolism, the homologs of these genes in other halobacterial species likely also have this function, allowing those species to utilize DHA. Halobacterial species without DHA kinase might also be capable of utilizing DHA if they possess a *glpK* gene, since our results indicate that glycerol kinase also plays a role in DHA metabolism. BLASTp results for *glpK* indicate that 82 halobacterial species have homologs, and 51 of these species do not have *dhaKLM* homologs. We suspect that these species are also able to metabolize DHA. Eighteen halobacterial species are missing DHA and glycerol kinase genes, suggesting that they cannot metabolize DHA. However, only three of those genomes, *Halovivax ruber, Natronobacterium gregoryi*, and *Natronomonas pharaonis*, are not in draft form, leaving open the possibility for a near universal distribution of DHA metabolism in Halobacteria.

The broad taxonomic distribution of DHA and glycerol kinase genes among the Halobacteria suggests two interwoven hypotheses: (i) DHA is a common carbon source in hypersaline environments and (ii) DHA metabolism is widespread among the Halobacteria. A study by Elevi Bardavid and Oren (2008) detailed the conversion by the halophilic bacterium *S. ruber* of glycerol to DHA, which was then used as a growth substrate by *Hqm. walsbyi*. They speculated that DHA could be a common carbon source due to incomplete oxidation of glycerol, and from it being an intermediate of glycerol synthesis in *Dunaliella*. Our data demonstrating the extensive incidence of DHA and glycerol kinase genes provides support for their hypothesis that DHA is a common carbon source, and extends it to include that many if not most Halobacteria are capable of metabolizing it. However, future research on DHA production and turnover rates, and analysis on strains we predict to have DHA metabolism is necessary to elucidate the significance of this substrate to hypersaline ecosystems and Halobacteria.

## Authors contributions

R. Thane Papke, Andrea M. Makkay, and Matthew Ouellette conceived the researched and wrote the manuscript. Andrea M. Makkay and Matthew Ouellette performed the research.

### Conflict of interest statement

The authors declare that the research was conducted in the absence of any commercial or financial relationships that could be construed as a potential conflict of interest.
